# Biodegradability of poly(lactic-co-glycolic acid) after femtosecond laser irradiation

**DOI:** 10.1038/srep27884

**Published:** 2016-06-15

**Authors:** Akimichi Shibata, Shuhei Yada, Mitsuhiro Terakawa

**Affiliations:** 1 School of Integrated Design Engineering, Keio University, 3-14-1, Hiyoshi, Kohoku-ku, Yokohama, 223-8522, Japan; 2 Department of Electronics and Electrical Engineering, Keio University, 3-14-1, Hiyoshi, Kohoku-ku, Yokohama, 223-8522, Japan

## Abstract

Biodegradation is a key property for biodegradable polymer-based tissue scaffolds because it can provide suitable space for cell growth as well as tailored sustainability depending on their role. Ultrashort pulsed lasers have been widely used for the precise processing of optically transparent materials, including biodegradable polymers. Here, we demonstrated the change in the biodegradation of a poly(lactic-co-glycolic acid) (PLGA) following irradiation with femtosecond laser pulses at different wavelengths. Microscopic observation as well as water absorption and mass change measurement revealed that the biodegradation of the PLGA varied significantly depending on the laser wavelength. There was a significant acceleration of the degradation rate upon 400 nm-laser irradiation, whereas 800 nm-laser irradiation did not induce a comparable degree of change. The X-ray photoelectron spectroscopy analysis indicated that laser pulses at the shorter wavelength dissociated the chemical bonds effectively, resulting in a higher degradation rate at an early stage of degradation.

Biodegradable polymers are attracting considerable interest in the field of biomedical applications because of their high mechanical strength, biocompatibility, and biodegradability. Poly(lactic acid) (PLA), poly(glycolic acid) (PGA), and their copolymer, poly(lactic-co-glycolic acid) (PLGA), have been approved by the FDA for clinical uses, such as bioabsorbable sutures^1^ and bone fixation devices^2^. The applications of biodegradable polymers also extend to the fields of drug delivery carriers^3^,^4^ and tissue scaffolding^5^,^6^. The biodegradation behavior is a key factor for such applications. Drug delivery carriers are required to gradually release an encapsulated drug as the biodegradable polymer carriers degrade. Biodegradable tissue scaffolds can provide sufficient space for cell growth with regard to cell spreading and proliferation^7^.

The biodegradation of a biodegradable polymer in a physiological environment proceeds *via* the hydrolysis of ester bonds in the presence of absorbed water. The degradation rate of a biodegradable polymer can be influenced by several factors, namely molecular weight, crystallinity, surface morphology, and surface hydrophilicity. Several methods have been proposed to control these factors, such as annealing^8^, copolymerization^9^, porous structure formation^10^, and light-based modification^11^,^12^,^13^,^14^. Light-based modification is a simple technique, which enables the modification of the chemical structures and morphologies of complex surfaces, even after molding, at a specific time. Short pulsed lasers have been applied to polymer processing, and have been used to accelerate the biodegradation of biodegradable polymers^13^,^14^. Hsu *et al.* reported that nanosecond ultraviolet laser irradiation accelerated the biodegradation of poly(L-lactic acid) (PLLA) due to a reduction in crystallinity associated with surface melting^13^. Farkas *et al.* experimentally investigated the effect of nanosecond ultraviolet laser irradiation, with different laser fluences and repetition rates, to poly(propylene fumarate) (PPF)^14^. To the best of our knowledge, only UV lasers have been applied, and the effect of laser wavelengths on the biodegradation of polymers is yet to be revealed because of their weak optical absorption for the visible to near infrared wavelengths.

The precise processing of optically transparent materials has been achieved using ultrashort pulsed lasers. Since many biodegradable polymers show higher transmittance for visible to near-infrared wavelengths, three dimensional structures can be fabricated *via* multiphoton absorption. Due to its extremely short pulse duration and high peak intensity with modest average power, the processing of biodegradable polymers without significant thermal modification has been achieved. Owing to these features, ultrafast laser processing has enabled the fabrications of microvessels^15^, stents^16^, and woodpile-shaped scaffolds consisting of biodegradable polymers^17^. Although nanoscale periodic surface structures have been fabricated on a PLLA surface^18^, and the effect of laser-induced surface microstructures on cell adhesion and proliferation have been widely investigated^19^,^20^,^21^, it should be noted that the underlying mechanism of interaction between the ultrashort laser pulse and polymer has yet to be elucidated in detail.

In this study, we demonstrated the difference in biodegradation of PLGA films after femtosecond laser irradiation, which is depending on the laser wavelengths. The effect of femtosecond laser irradiation on the surface morphology and chemical structure of PLGA films was investigated *via* X-ray photoelectron spectroscopy (XPS), Fourier transform infrared spectroscopy (FTIR), gel permeation chromatography (GPC), X-ray diffraction (XRD) analyses and scanning electron microscopy (SEM) observation.

## Results

### Chemical and structural analysis of PLGA films before and after femtosecond laser irradiation

**Figure 1:**
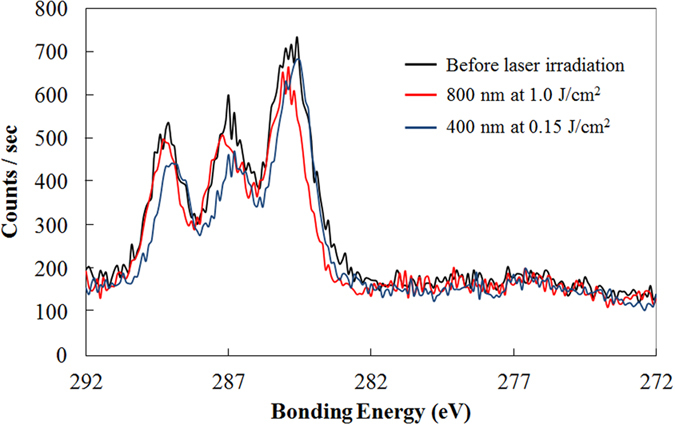
C1s narrow scan XPS spectra of the surface of PLGA films before and after laser irradiation. Black, red and blue lines indicate before laser irradiation, after laser irradiation with 800 nm at 1.0 J/cm^2^, and after laser irradiation with 400 nm at 0.15 J/cm^2^, respectively. The scanning speed was 30 μm/s.

**Figure 2:**
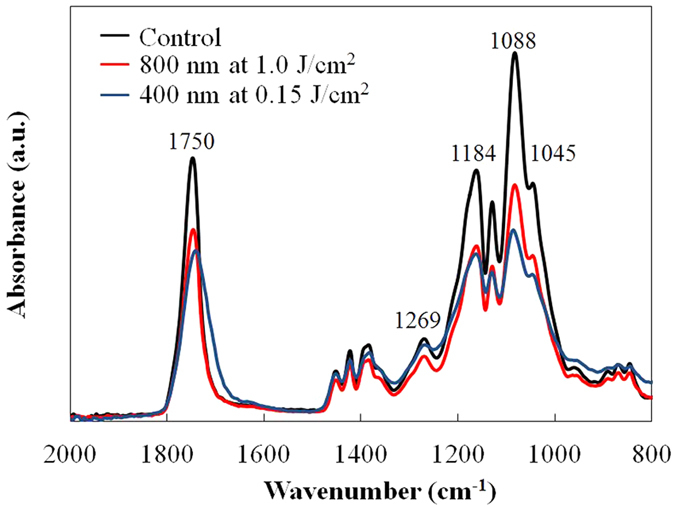
FTIR spectra of the surface of PLGA films before and after laser irradiation. Black, red, and blue lines indicate before laser irradiation, after laser irradiation with 800 nm at 1.0 J/cm^2^, and after laser irradiation with 400 nm at 0.15 J/cm^2^, respectively. The laser scanning speed was 30 μm/s.

**Figure 3:**
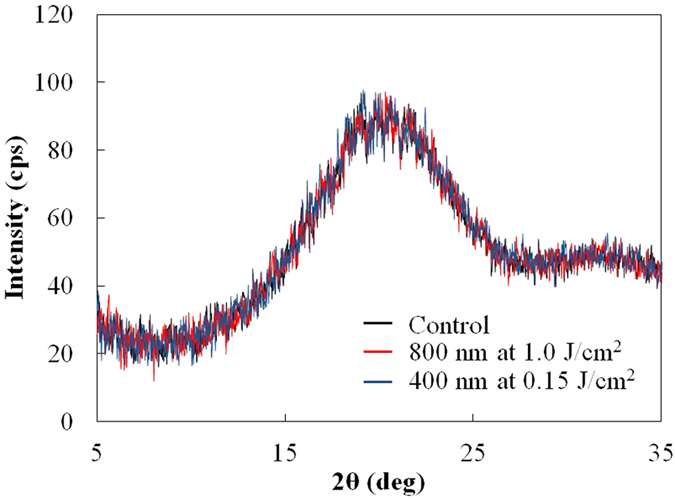
XRD spectra of PLGA films before and after laser irradiation. Black, red, and blue lines indicate before laser irradiation, after laser irradiation with 800 nm at 1.0 J/cm^2^, and after laser irradiation with 400 nm at 0.15 J/cm^2^, respectively. The laser scanning speed was 30 μm/s.

Figure 1 shows the C1s narrow scan XPS spectra of the PLGA film surface before and after laser irradiation. The spectra consist of three peaks (bonding energies of 285 eV, 287 eV, and 289 eV), which are attributable to the carbon in the alkyl group (–CH_3_), neighboring carbon in the C–O bond, and carbon in the ester bond (O–C=O), respectively^22^. As shown in Fig. 1, 287 eV and 289 eV peaks reduced in height following laser irradiation, especially with 400 nm laser irradiation. This indicates that the quantity of C–O bonds and C=O bonds decreased because of the photolysis induced by the laser irradiation. As measured by XPS, the O_1s_ to C_1s_ ratios were 0.758, 0.785, and 0.634 prior to laser irradiation, following laser irradiation at wavelengths of 800 nm and 400 nm, respectively. The O/C ratio did not significantly change with 800 nm laser irradiation; however, the 400 nm laser irradiation resulted in a significant decrease in the ratio, which is consistent with the results of a previous study using a nanosecond laser^13^. Figure 2 shows the FTIR spectra of the PLGA film surface before and after laser irradiation. Following the laser irradiation, the peaks attributed to –C=O carbonyl stretching (1750 cm^−1^) and –C–O– stretching (1184 cm^−1^ and 1088 cm^−1^)^23^ decreased, especially in the case of the 400 nm-laser irradiation. This indicates that chemical bond dissociation occurred not only at the film surface but also within the film. In addition, using GPC, we measured the average molecular weight of the PLGA before and after laser irradiation. Under control condition, and laser irradiation at wavelengths of 800 nm and 400 nm, the weight average molecular weights (*M*_w_) of the PLGA were 27784 g/mol, 28064 g/mol, and 26785 g/mol, respectively. The number average molecular weights (*M*_*n*_) obtained under the aforementioned conditions were 17157 g/mol, 17402 g/mol, and 15789 g/mol, respectively. The GPC analysis showed that the average molecular weight decreased following 400 nm-laser irradiation. Since the GPC analysis measures the entire film, there was probably a more significant decrease in the molecular weight of the PLGA on the surface layer following the 400 nm-laser irradiation. Figure 3 shows the results of the XRD analysis performed on the PLGA film before and after laser irradiation. There was no significant difference between the XRD spectra observed before and after laser irradiation, indicating that the reduction in the PLGA crystallinity is limited.

### Morphology transition of laser ablation craters during degradation

**Figure 4:**
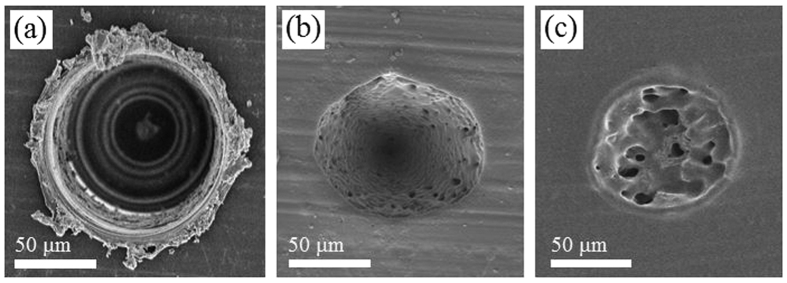
SEM images of the craters formed on PLGA films. Scale bars represent 50 μm. (**a**) The crater formed by mechanically milling. (**b**) The laser ablation crater under the condition of 800 nm, 1.0 J/cm^2^, 15000 pulses. (**c**) The laser ablation crater under the condition of 400 nm, 0.15 J/cm^2^, 15000 pulses.

Figure 4 shows SEM images of the craters on the PLGA films, which were formed by mechanical milling and laser irradiation at wavelengths of 800 nm and 400 nm. The laser irradiation conditions were selected to fabricate a laser ablation crater with a diameter of approximately 100 μm. A burr was observed on the edge of the crater that was formed by mechanical milling (Fig. 4a). The surface of the laser ablation crater formed with a laser wavelength of 800 nm possessed rough and small pores (Fig. 4b). Probable evidence of melting was observed on the periphery of the laser ablation crater formed with a laser wavelength of 400 nm (Fig. 4c).

**Figure 5:**
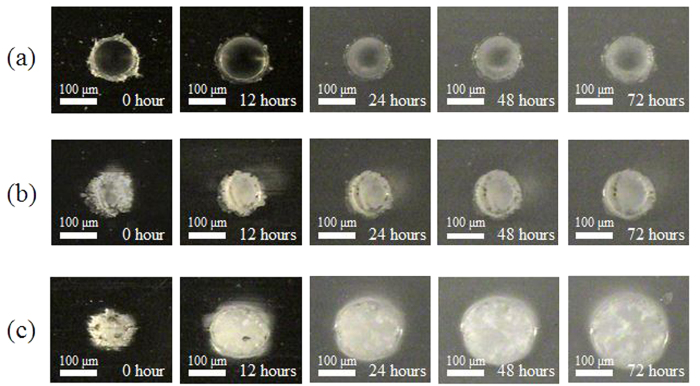
Digital microscopic images of the craters formed on PLGA films. The time shown in each figure indicate the time of the samples immersed in PBS at 37 °C. Scale bars represent 100 μm. (**a**) The crater formed by mechanically milling. (**b**) The laser ablation crater under the condition of 800 nm, 1.0 J/cm^2^, 15000 pulses. (**c**) The laser ablation crater under the condition of 400 nm, 0.15 J/cm^2^, 15000 pulses.

Figure 5 shows digital microscopic images of the fabricated craters with regard to time. Compared with the mechanically milled crater, the craters formed by laser ablation exhibited a turbid appearance prior to the biodegradation test. The size of the mechanically milled crater remained relatively unchanged, even following 72 h of immersion. The crater formed by laser ablation at a laser wavelength of 800 nm exhibited slight expansion following 48 h of immersion. In contrast with the laser ablation crater formed with a laser wavelength of 800 nm, the laser ablation crater formed with a laser wavelength of 400 nm expanded drastically. As shown in Fig. 5c, the expansion was obvious, even following 12 h of immersion. The expansion continued for 72 h; at this instance, we terminated the measurements. It is also noteworthy that the digital microscopic images appeared more turbid as the immersion period was extended. This could be attributed to the change in the crystallinity, because an amorphous site degrades at a faster rate than a crystalline site^24^.

**Figure 6:**
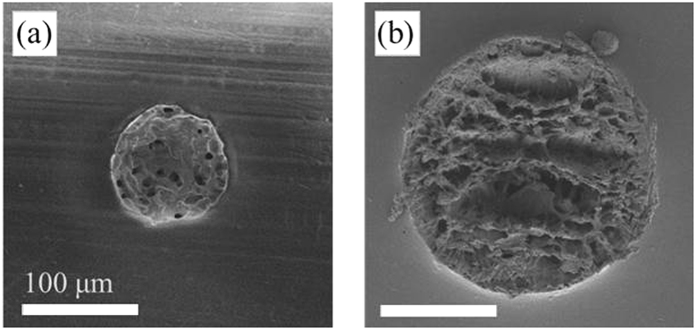
SEM images of the laser ablation craters under the condition of 400 nm, 0.15 J/cm^2^, 15000 pulses. Scale bars represent 100 μm. (**a**) Immediately after the laser irradiation, (**b**) 72 hours after the immersion, the same sample used for (**a**).

To confirm that the size of the turbid site observed with the digital microscope was consistent with that of the crater before and 72 h after degradation, we observed the crater formed by laser ablation at a wavelength of 400 nm using SEM (Fig. 6). The sizes of the craters formed by laser ablation, in Fig. 6, correlate well with the results shown in Fig. 5, which were recorded under the same conditions.

**Figure 7:**
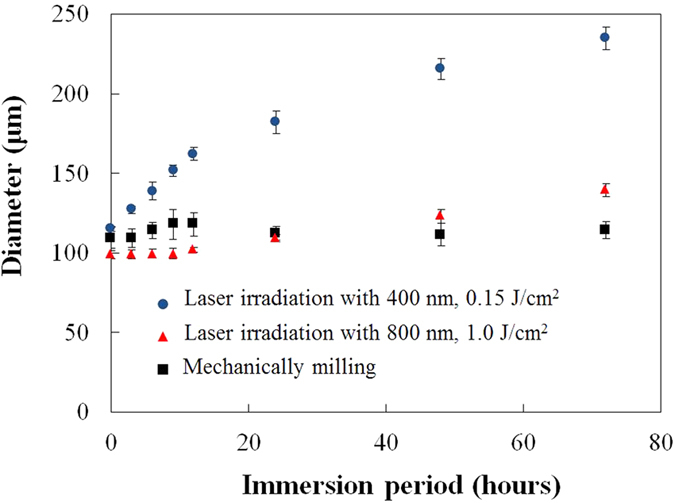
The diameters of the craters formed on PLGA films. The time represents immersion time after craters formation. Black closed squares (▪), red triangles (▴), blue closed circles (⦁) indicate the diameters of the crater formed by mechanically milling, the laser ablation craters with 800 nm, 1.0 J/cm^2^, 15000 pulses, the laser ablation craters with 400 nm, 0.15 J/cm^2^, 15000 pulses, respectively. All values are expressed as means ± standard deviation (SD).

The diameters of five different turbid sites (N = 5) were measured for each immersion period (0, 3, 6, 9, 12, 24, 48, 72 h) for quantitative and statistical evaluation (Fig. 7). Over the immersion period of 72 h, the diameters of the mechanically milled craters were relatively constant, at approximately 100 μm. The size of the craters formed by laser ablation at a wavelength of 800 nm remained relatively unchanged over an immersion period of 12 h, and subsequently the crater started to gradually expand. Following 72 h, the average diameter was 139 ± 4 μm. The craters that were formed by laser ablation at a wavelength of 400 nm exhibited comparably rapid expansion over an immersion period of 12 h. The expansion of the crater diameter continued to expand over 72 hours of immersion. Following immersion periods of 12 h and 72 h, the average diameters of the craters formed by laser ablation at a wavelength of 400 nm were 162 ± 4 μm and 235 ± 7 μm, respectively.

### Water absorption and weight reduction during degradation

**Figure 8:**
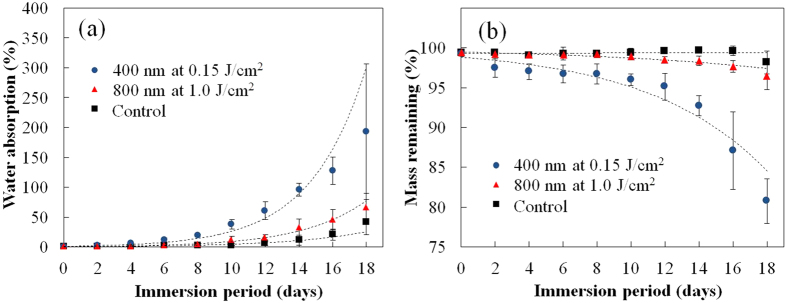
(**a**) Water absorption and (**b**) Mass remaining of PLGA films during degradation. Closed squares (▪), red triangles (▴), and blue closed-circles (⦁) indicate the control PLGA films, PLGA films irradiated with 800 nm at 1.0 J/cm^2^, and PLGA films irradiated with 400 nm at 0.15 J/cm^2^, respectively. The scanning speed was 30 μm/s. All values are expressed as means ± SD. The dotted lines indicate exponential fitted curve of the given dataset.

We measured the water absorption and change in mass of the PLGA films to investigate the degradation characteristics in detail. The water absorption of the laser-irradiated PLGA films was greater than that of the control (non-irradiated) PLGA films, as shown in Fig. 8a. The 400-nm laser-irradiated PLGA films exhibited enhanced and faster water absorption compared with the 800-nm-laser-irradiated films, especially after an immersion period of 8 days. The water absorption curves were fitted by exponential equations, which can be expressed as follows:


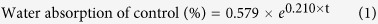



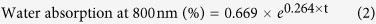



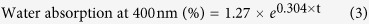


where *t* indicates the number of days of immersion. The exponential coefficient in the equation (3) is highest among three samples. As shown in Fig. 8b, following 12 days of immersion, there was a significant reduction in the mass of the 400 nm-laser-irradiated samples. Following 18 days of immersion, the mass of the 400 nm-laser-irradiated samples decreased to 80.7% of the initial mass. Moreover, following 18 days of immersion, the masses of the control sample and the 800 nm-laser-irradiated samples decreased slightly to 98.17% and 96.49%, respectively. The mass remaining profiles of the PLGA were also fitted with exponential equations as shown in Fig. 8b. The equations are expressed as follows:


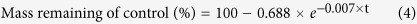



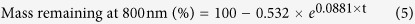



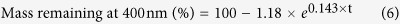


**Figure 9:**
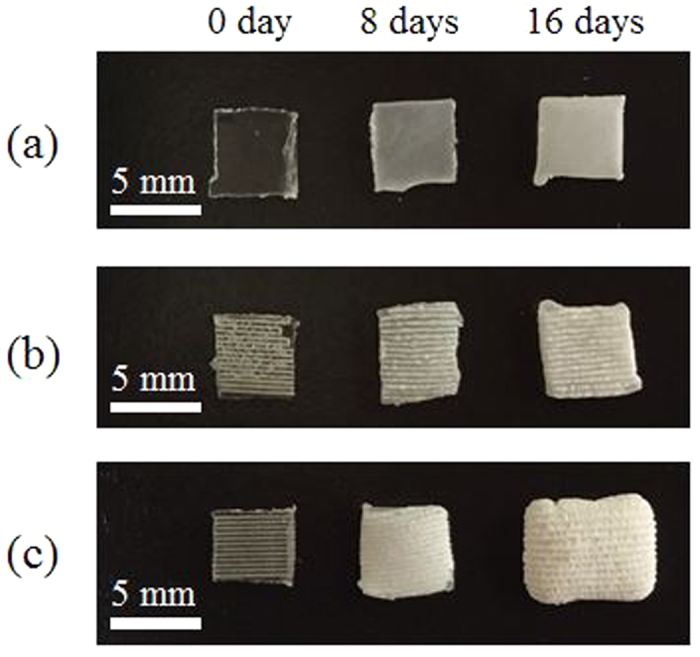
The photographs of PLGA films before and after 8 or 16 days of immersion. Each sample was dried in vacuum for 6 days. Scale bars represent 5 mm. (**a**) Control PLGA films. (**b**) PLGA films irradiated with 800 nm at 1.0 J/cm^2^. (**c**) PLGA films irradiated with 400 nm at 0.15 J/cm^2^. The scanning speed was 30 μm/s.

Considering the decay curves of the remaining mass, after 2 days of immersion, a substantial decrease in mass was observed for 400 nm-laser-irradiated samples, which can be attributed to burst release upon laser irradiation, followed by the higher degradation rate. The morphology of the PLGA films also changed during degradation. Figure 9 shows photographs of the PLGA films before (0 days) and after degradation (8 or 16 days). All the samples developed a turbid appearance because of an increase in crystallinity, which was induced by the degradation. Following 8 days of immersion, the control samples and 800 nm-laser-irradiated samples appeared semitransparent; however, the 400 nm-laser-irradiated samples appeared completely turbid.

## Discussion

Ultrafast laser processing has been widely studied with regard to various materials, including polymers, because it can be used to fabricate precise structures with extremely small heat affected zone (HAZ). Although the importance of biodegradability, which affects biocompatibility^25^ and sustainability^26^, has been discussed in previous studies involving the ultrafast laser processing of biodegradable polymers^15^,^16^,^17^,^18^,^19^,^20^,^21^, the effect of ultrashort pulsed laser irradiation on the biodegradability of biodegradable polymers has, to the best of our knowledge, not been reported.

The XPS and FTIR analyses of the PLGA films showed that there was a reduction in the quantity of C–O bonds and C=O bonds following laser irradiation, especially at a wavelength of 400 nm (Figs 1 and 2). The dissociation of chemical bonds is due to the Norrish type II reaction^11^. The Norrish type II reaction is a chemical reaction that is induced by the photoexcitation of ester bonds *via* the *n* − π* transition, which results in the dissociation of C–O bonds. Photon energy of approximately 4 eV is required to induce the *n* − π* transition^27^. The laser wavelengths of 800 nm and 400 nm have photon energies of 1.55 eV and 3.10 eV, respectively. Therefore, the electrons in the ester bonds could be excited *via* three-photon absorption and two-photon absorption, respectively. Compared with three-photon absorption, two-photon absorption is more likely to occur; therefore, the *n* − π* transition is likely to occur *via* 400 nm-laser irradiation, which results in dissociation of the chemical bonds in the PLGA films. In addition, the degree of melting observed around the laser ablation craters could be attributed to excess photon energy. The excessive energy of two-photon absorption after *n* − π* transition with 400 nm is greater compared with that with 800 nm. Moreover, the excess energy would be partially converted into thermal energy *via* vibrational relaxation. Therefore, this explains why traces of melting were observed at the periphery of the laser ablation craters formed with a laser wavelength of 400 nm (Fig. 4c).

As shown in Figs 7 and 8, the 400 nm-laser-irradiated PLGA films significantly degraded compared with the non-irradiated PLGA films. In a physiological environment, the biodegradation process is divided into two stages. In the first stage, the biodegradable polymer is hydrolyzed by water, resulting in a reduction of the molecular weight. In the second stage, the polymer with a low molecular weight within the film diffuses in the water, resulting in a reduction of mass. Thus, water absorption is essential for the degradation of biodegradable polymers. The change in the water absorption can be attributed to two effects that are induced by light-polymer interactions; (i) a decrease in crystallinity, and (ii) a decrease in molecular weight^11^,^12^,^13^,^14^.

The first factor affecting the water absorption is the decrease in crystallinity. The degradation rate of a crystalline site is generally slower than that of an amorphous site because of the inability of water to diffuse into a highly ordered crystalline site^24^. Therefore, a decrease in crystallinity accelerates the degradation. Hsu *et al.* reported that a PLLA film irradiated with a nanosecond laser shows a higher rate of the mass loss than a control PLLA film^13^. They suggested that the decrease in crystallinity, due to the melting, enhanced the water diffusion, resulting in a higher degradation rate. As shown in Fig. 4c, the traces of melting can be observed in the laser ablation crater formed with a laser wavelength of 400 nm. A significant part of the melted site consists of an amorphous phase, where the crystallinity is lower and faster degradation rate compared with the non-irradiated site. On the other hand, the HAZ was negligible around the crater formed by laser ablation with a wavelength of 800 nm (Fig. 4b). This result suggests that the decrease in crystallinity around the laser irradiated spot with 800 nm is limited. Therefore, the degradation rate did not change significantly.

The second factor that affects the water absorption is the decrease in molecular weight owing to the photolysis of chemical bonds. Polymer molecules can diffuse out of a bulk polymer film when the molecular weight of each polymer chain is lower than a critical value^26^. The decrease in molecular weight that was induced by the light irradiation shortened the hydrolysis period required to attain the critical value, resulting in a faster mass decrease. In addition, the dissociation of bonds results in the acceleration of hydrolysis since the degradation products, including hydrophilic carboxylic acid, increase the degree of water absorption^28^. The diameter of the laser ablation crater that was formed with a laser wavelength of 400 nm expanded more rapidly than that with 800 nm (Fig. 7). Since the applied laser pulses have non-flat beam profile and the laser spot size of 100 μm is a value of FWHM, the practical area of interaction is larger than 100 μm. It is possible the degradation rate varies for interaction areas with lower intensity; however, the results show that the crater formed by laser ablation with a wavelength 400 nm even expands outside beyond the area that was irradiated. This result suggests that 400 nm-laser irradiation not only accelerates the degradation of the irradiated spot but also induces propagates degradation originating from the laser spot. As shown in Fig. 8, the 400 nm-laser-irradiated PLGA films show the higher degree of water absorption and a faster mass decrease compared with the 800 nm-laser-irradiated PLGA films. These results are consistent with those of the XPS analysis, which indicates that the photolysis of the chemical bonds within 400 nm-laser-irradiated samples is more significant compared with that of 800 nm-laser-irradiated samples (Fig. 1). Tsuji *et al.* reported that the UV light irradiation of PLLA results in a decrease in molecular weight for both amorphous and crystalline sites^12^. Considering the significant decrease in molecular weight due to the 400 nm-laser irradiation, it is acceptable that the PLGA films degrade faster when the molecular weight decreases at the crystalline sites. Moreover, following 800 nm-laser-irradiation, the degradation rate did not significantly change. This can be explained by the slight decrease in molecular weight due to the less chemical bond dissociation.

According to the discussion above, there are two possible hypotheses that explain the difference between the degradation rates of the PLGA films treated with different laser wavelengths; the amorphization and the decrease in molecular weight. However, considering that the crystallinity was relatively constant following laser irradiation (Fig. 3) and that significant chemical bond dissociation occurred following 400 nm-laser irradiation (Fig. 1), the difference in the degradation rate can be attributed to the decrease in molecular weight induced by laser irradiation. The degradation rates of PLGA films can also be observed by the color change of PLGA films. During the degradation process, the amorphous sites degrade rapidly compared with the crystalline sites and thus the crystallinity of the film eventually increases^24^. Because the light scattering in crystalline sites is stronger than that in amorphous sites due to the higher refractive index, these sites show much more turbid appearance. As shown in Fig. 9, the 400 nm-laser-irradiated PLGA films became turbid appearance more rapidly compared with the other films.

## Conclusion

We demonstrated that laser wavelengths affect the biodegradability of the polymer following femtosecond laser irradiation. A smaller HAZ was observed at the periphery of the laser ablation crater formed with 800 nm-laser irradiation compared with that with 400 nm-laser irradiation. The results of the degradation tests suggest that the degradation rate of the 400 nm-laser-irradiated PLGA film was much greater than that of the 800 nm-laser-irradiated PLGA films. By considering the XPS, FTIR, and GPC results, in the case of the femtosecond laser irradiation at a wavelength of 400 nm, the significant acceleration of the biodegradation could be attributed to the decrease in molecular weight induced by the chemical bond dissociation. For the first time, our study revealed that the degradation rate of a biodegradable polymer depends on the laser wavelength. In addition, our study shows that femtosecond laser processing has potential to control the degradation and sustainability of a structure following its fabrication.

## Methods

### Sample preparation and characteristics

PLGA films with lactic to glycolic acid ratio of 50:50 mol% were obtained from BMG Inc. (Kyoto, Japan). Films were prepared by a solvent casting method. Weight average molecular weight (*M*_w_), number average molecular weight (*M*_n_), glass transition temperature (*T*_g_) were 27648 g mol^−1^, 16919 g mol^−1^, 44.0 °C, respectively. The thickness of the film was 1 mm. All data was provided by BMG Inc.

### Femtosecond laser irradiation

Linearly polarized femtosecond laser pulses at 800 nm (fundamental wave) or 400 nm (second harmonic wave) in central wavelengths from a Ti:sapphire chirped pulse amplification (CPA) laser system (Libra, Coherent, Inc., California, USA) were focused onto a surface of a PLGA film at normal incidence using a plano-convex lens (focal length 200 mm) in air. The laser was operated in an external trigger mode, which provides the desired number of pulses at a repetition rate of 1 kHz. Pulse width after the CPA was ~100 fs at 800 nm wavelength measured with a multishot autocorrelator.

### Chemical and structural analyses of PLGA films

The modification of the chemical structure of the PLGA surface before and after the laser irradiation was analyzed by XPS (JPS-9010, JEOL, Tokyo, Japan) and by FTIR (ALPHA FT-IR Spectrometers, BRUKER, Massachusetts, USA). The weight average molecular weight (*M*_w_) and the number average molecular weight (*M*_n_) were determined through GPC equipped with a refractive index detector (SHIMADZU, Kyoto, Japan). The modification of the crystallinity of the PLGA surface before and after the laser irradiation was analyzed by XRD (D8 DISCOVER, BRUKER, Massachusetts, USA). Samples for the analyses were prepared by scanning focused femtosecond laser pulses on the PLGA film. The laser fluences at 800 nm and 400 nm were 1.0 J/cm^2^ and 0.15 J/cm^2^, respectively, so as to fabricate the laser ablation craters with the same diameter. The scanning speed and the spot diameter were 30 μm/s and 150 μm, respectively.

### Measurement of crater diameter during degradation

The biodegradability of PLGA after femtosecond laser irradiation was evaluated morphologically by observing the change in laser ablation crater diameters. Laser fluences and number of pulses were carefully selected to fabricate a laser ablation crater of 100 μm in diameter to minimize the initial difference in the diameters with 800 nm and 400 nm wavelengths. At 800 nm in wavelength, 15000 femtosecond laser pulses were irradiated at the laser fluence of 1.0 J/cm^2^. At 400 nm wavelength, 15000 femtosecond laser pulses at the laser fluence of 0.15 J/cm^2^ were irradiated on the surface of PLGA films. For comparison, a dented hole of 100 μm diameter with 100 μm in depth was also mechanically milled with a fraise (KE-55, Makino Milling Machine Co., Ltd, Tokyo, Japan) on the surface of PLGA films. Each sample was fully immersed in a 2 mL phosphate-buffered saline (PBS) with pH of 7.4 in a vial. The vials were gently placed in a water bath maintained at 37 °C. Samples were taken out and the surface of a film was observed with digital microscope (MS-100, Asahikogaku, Tokyo, Japan) after the elapse of immersion time for 3, 6, 9, 12, 24, 48, 72 hours. Scanning electron microscopy was also performed.

### Measurement of water absorption and mass change during degradation

PLGA films of 5 × 5 mm^2^ were used. Each sample was prepared by scanning focused femtosecond laser pulses (scanning speed of 30 μm/s, spot size of 150 μm) on one side of the PLGA film. The laser fluences at 800 nm and 400 nm were 1.0 J/cm^2^ and 0.15 J/cm^2^, respectively, which are the same conditions for the sample preparation for the XPS analysis. Before degradation, initial mass (*m*_ini_) of each sample was measured with an electronic balance immediately after the laser irradiation to measure the initial mass of the films (*m*_ini_). Samples were fully immersed in 2 mL phosphate-buffered saline (PBS) with pH of 7.4 in vials. The vials were gently placed in a water bath maintained at 37 °C. PBS was replaced every 2 days in order to avoid a change in pH of PBS. The films were removed from the vials and weighed immediately to obtain the wet mass (*m*_wet_). Films were then washed gently with distilled water and dried in vacuum for 6 days to obtain the dry mass (*m*_dry_). This procedure was repeated with different immersion period (2 to 18 days). The water absorption and the mass remaining of the films were calculated by following equations.


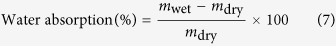



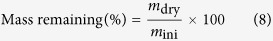


## Additional Information

**How to cite this article**: Shibata, A. *et al.* Biodegradability of poly(lactic-co-glycolic acid) after femtosecond laser irradiation. *Sci. Rep.* **6**, 27884; doi: 10.1038/srep27884 (2016).
